# Computational imaging reveals shape differences between normal and malignant prostates on MRI

**DOI:** 10.1038/srep41261

**Published:** 2017-02-01

**Authors:** Mirabela Rusu, Andrei S. Purysko, Sadhna Verma, Jonathan Kiechle, Jay Gollamudi, Soumya Ghose, Karin Herrmann, Vikas Gulani, Raj Paspulati, Lee Ponsky, Maret Böhm, Anne-Maree Haynes, Daniel Moses, Ron Shnier, Warick Delprado, James Thompson, Phillip Stricker, Anant Madabhushi

**Affiliations:** 1 Department of Biomedical Engineering, Case Western Reserve University, 10900 Euclid Avenue, Cleveland, Ohio, 44106, USA; 2 Section of Abdominal Imaging and Nuclear Radiology Department, Imaging Institute, Cleveland Clinic, 9500 Euclid Avenue, Cleveland OH 44195, USA; 3 University of Cincinnati Medical Center, 234 Goodman Street, Cincinnati, Ohio, 45267, USA; 4 University Hospitals Cleveland Medical Center and Case Western Reserve University, 11100 Euclid Avenue, Cleveland, Ohio, 44106, USA; 5 Garvan Institute of Medical Research, The Kinghorn Cancer Centre, 384 Victoria St, Darlinghurst, New South Wales, 2010, Australia; 6 Radiology Department, Spectrum Medical Imaging, 13-15 Silver St, Randwick, New South Wales, 2031, Australia; 7 Southern Radiology, Neuroscience Research Australia, Barker St & Easy St, Randwick, New South Wales, 2031, Australia; 8 Douglass Hanly Moir Pathology, 14 Giffnock Ave, Macquarie Park, New South Wales, 2113, Australia; 9 St. Vincent’s Prostate Cancer Center, 390 Victoria St, Darlinghurst, New South Wales, 2010, Australia; 10 School of Medicine, University of New South Wales, 18 High St, Kensington, New South Wales, 2052, Australia

## Abstract

We seek to characterize differences in the shape of the prostate and the central gland (combined central and transitional zones) between men with biopsy confirmed prostate cancer and men who were identified as not having prostate cancer either on account of a negative biopsy or had pelvic imaging done for a non-prostate malignancy. T2w MRI from 70 men were acquired at three institutions. The cancer positive group (PCa+) comprised 35 biopsy positive (Bx+) subjects from three institutions (Gleason scores: 6–9, Stage: T1–T3). The negative group (PCa−) combined 24 biopsy negative (Bx−) from two institutions and 11 subjects diagnosed with rectal cancer but with no clinical or MRI indications of prostate cancer (Cl−). The boundaries of the prostate and central gland were delineated on T2w MRI by two expert raters and were used to construct statistical shape atlases for the PCa+, Bx− and Cl− prostates. An atlas comparison was performed via per-voxel statistical tests to localize shape differences (significance assessed at p < 0.05). The atlas comparison revealed central gland hypertrophy in the Bx− subpopulation, resulting in significant volume and posterior side shape differences relative to PCa+ group. Significant differences in the corresponding prostate shapes were noted at the apex when comparing the Cl− and PCa+ prostates.

Multi-parametric magnetic resonance imaging (MRI) plays an essential role in the management of prostate cancer (PCa), improving localization and local staging of the disease^1^,^2^. In addition to providing structural and functional images of the prostate, prostate MRI has also revealed differences in cancers based on their localization in the anatomic subregions of the prostate^3^. Cancers localized in the peripheral zone are more frequent^4^ and have a different MRI appearance^3^ compared to the cancers localized in the central (CZ) or transitional zones (TZ), the TZ and CZ together constituting the central gland (CG). Recent guidelines for scoring of PCa incorporate different recommendations for cancers localized in the transitional zone or in the peripheral zone^2^,^4^. Furthermore, recent quantitative studies^5^,^6^ have identified different textural imaging signatures that appear to characterize cancers localized in CG or PZ.

The goal of the current study is to characterize the influence of prostate cancer on the shape of the entire prostate and CG. Unlike previous studies^7^ that assessed volume changes in the prostatic subregions, our study focuses on evaluating changes in the 3D shape of the prostate and CG outlined on T2-weighted (T2w) MRI. T2w MRI has been shown to best highlight structural attributes of the prostate and prostate cancer. Such characterization might provide quantitative measures to facilitate diagnosis or outcome prediction.

Shape variations in anatomic regions have been often investigated in population studies^8^,^9^,^10^,^11^,^12^, where the focus is on characterizing the utility of shape differences to predict disease presence and prognosis. For instance, the hippocampus shape has been shown to be correlated with Alzeimer’s disease progression and was suggested as a potential quantitative imaging biomarker to predict dementia progression^10^. Moreover, population analysis via statistical atlases are of particular utility as they combine information from multiple subjects into one unified representation^13^,^14^,^15^, thus facilitating quantitative comparisons across different populations. Various atlas approaches have been utilized to study the brain (e.g. ref. 14) and, more recently, the prostate^15^,^16^.

The current work tests the hypothesis that PCa induces shape changes in the anatomy of the prostate. Specifically, we investigate the utility of statistical shape atlases to characterize differences between subjects with or without prostate cancer, particularly focusing on the shape of the prostate boundary and CG on pelvic T2w MRI scans acquired at multiple institutions. Owing to the difficulty in locating and delineating the boundaries of TZ and CZ, we focus in this study on studying shape differences of the CG. Our study included 70 subjects from three institutions with 3 Tesla (T) MRI acquired using either a surface or an endorectal coil. The PCa positive group (PCa+) included 35 biopsy positive (Bx+) subjects from all the institutions. The prostate cancer negative (PCa−) included 24 biopsy negative (Bx−) subjects from two institutions and 11 prostates from subjects suspicious for rectal cancer (and hence had a pelvic imaging scan done), without clinical indication (Cl−) of PCa at the third institution.

To characterize anatomic shape differences, we applied the anatomically constrained registration previously described by us in ref. 15 and created statistical shape atlases of the prostate and CG. The anatomically constrained registration was chosen for atlas construction, since it explicitly ensures accurate alignment of the prostate and CG between multiple subjects. Furthermore, we expanded the approach to enable atlas comparisons which allows us to identify shape differences between different subpopulations. Other statistical shape atlas construction methods^17^ only use organ shape during atlas construction and hence are less likely to provide an accurate representation of CG, since the shape of CG tends to vary greatly between subjects.

Manual delineations of the prostate and the CG were obtained from two expert raters using the T2w MRI protocol as reference and were utilized when creating the atlases. All subjects in a subpopulation (PCa+ or PCa−) were elastically aligned relative to each other in a way that kept the prostate as well as CG in alignment between subjects during the atlas construction. For each subpopulation, the framework yielded an atlas with multiple constituent sub-atlases or compartments, the prostate statistical and the CG statistical shape atlases^15^. The resulting atlases for each of the PCa+ and PCa− population, reflects the intensity and shape of the anatomic regions across each of the individual subpopulations. Similar to ref. 16, per-voxel statistical comparisons between the two subpopulations were performed to identify shape differences between PCa+ and PCa− subjects.

A series of comparisons were performed between the PCa+ and PCa− atlases. First, we assessed the shape differences between PCa+ and PCa− subjects. In the second experiment, we stratified the PCa+ and PCa− subpopulations by institution and investigated the variability of shape differences between Bx+ and Bx− subjects, as well as Bx+ and Cl− prostates. The Cl− subjects constitute a different clinical population than the Bx−, since the latter represents a population of men with elevated prostate specific antigen (PSA) and other clinical indications that warranted a prostate biopsy.

## Results

### Brief Review of Concepts and Commonly Used Notation and Terminology

The volume of anatomic regions in the prostate, e.g. entire gland, refers to the numerical value resulting from adding the volumes of all image voxels within the T2w MRI scan of the prostate. Voxels are cuboids, and their volume in milliliters (ml) is computed by multiplying the in-plane resolution along X and Y, respectively, with the spacing between slices.

Comparing prostate shapes between subpopulations is done once all prostate MRIs are mapped into a common space, also known as common coordinate frame. The atlas is constructed in this space, and serves as reference space for the mapping of all prostate MRI scans from within a given subpopulation. Specifically, each subject MRI is transformed to match the atlas, the atlas serving as a fixed, registration reference (see details in the Material and Methods section).

The PCa+ subpopulation is denoted by *C*^+^ while the PCa− subpopulation is represented as *C*^−^. The MRI scans were acquired at three different institutions, denoted by subscripts 1–3, resulting in subpopulations 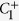, 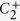, 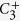, 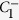, 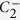 and 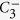.

Our study included delineations of CG and prostate from two expert readers. To assess the inter-rater effect on the observed differences we constructed 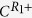 and 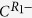 atlases using prostate and CG delineations obtained from rater 1. Similarly, the delineations of rater 2 were utilized to construct 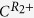 and 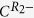. When not explicitly referencing the raters, we used *C*^+^ and *C*^−^ in place of 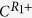 and 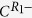. Moreover, for simplicity we constructed the subpopulations atlases, 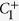, 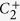, 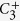, 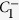, 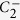 and 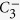, using only the delineations of rater 1.

### Comparing PCa+ and PCa− subpopulations prior to atlas construction

The volumes of the prostate and CG were consistent for the patients across 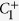, 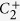, 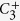 (Table 1). However, larger variability is observable in the volumes of the prostate and CG in the *C*^−^ subpopulation, since the Bx− subjects in 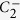 and 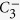 have mean prostate and CG volumes that are greater than twice of the Cl− subjects in 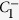 (Table 1). Moreover, Bx− subjects from both 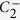 and 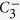 have prostate and CG volumes that are statistically significantly different compared to the Bx+ subjects in 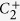 and 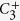, respectively. However, such differences were not observed in the 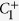 subpopulation, where Bx+ subjects had prostate and CG volumes that were comparable to the Cl− subjects. This may be, at least partially, due to the fact that Bx− subjects tend to have enlarged prostates, possibly due to the elevated PSA concentration levels that warranted the biopsy.

The latter observations hold independent of the expertise of the raters who outlined the CG and prostate regions (Table 1). The prostate and CG volumes observed with Rater 1 are similar compared to Rater 2. Further, no statistically significant differences in prostate and CG volumes were observed when comparing the delineations of the two raters for subjects within *C*^+^, *C*^−^, 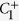, 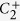, 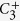, 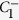, 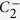 or 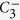. Moreover, the *κ* inter-rater agreement coefficient was determined to be 0.98 ± 0.03 for the prostate and 0.97 ± 0.05 for the CG, when comparing the two raters annotations across the 70 subjects included in the study. Such large *κ* coefficients suggest a good agreement between raters. Note that the *κ* coefficient varies between −1 and 1, where −1 corresponds to no overlap while 1 reflects a perfect overlap.

The comparison of the *C*^+^ and *C*^−^ subpopulations prior to atlas construction revealed volume differences, which needed to be corrected for in order to characterize shape differences. Indeed, a volume increase due to conditions such as benign prostatic hyperplasia, may cause a shape change in the CG and prostate. Hence we attempted to normalize for volumetric differences so that we could determine whether the observed shape differences were on account of volume difference or on account of the presence of disease.

### Comparing prostate and central gland volumes between PCa+ and PCa− subpopulations

The atlas construction framework was able to successfully correct for the variability in the volume of the prostate and the CG in the subpopulations (Table 2). This is reflected in the reduced standard deviation of the prostate and CG volumes compared to their variability prior to atlas construction (Table 1). The reduced standard deviation is observed independent of the raters.

The approach enabled the successful alignment of the prostate boundary between the *C*^+^ and *C*^−^ subpopulations. No statistically significant differences in prostate volumes were found between *C*^+^ and *C*^−^ subpopulations following the alignment (p value > 0.05). This lack of statistical significance in prostate shape differences between PCa+ and PCa− subpopulations was observed independent of the rater that delineated the prostate. These results appear to suggest that the atlas construction was able to correct for prostate volume differences between *C*^+^ and *C*^−^, and also differences on account of variations in MRI acquisition across institutions, i.e. 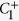 and 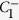, 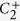 and 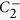, 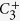 and 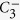. Also the atlas construction preserved the relative prostate and CG volumes in the subpopulations as statistically significant differences remain observable in the CG volumes between Bx+ and Bx− subpopulations, in *C*_2_ and *C*_3_, respectively. Consistent volume of both the prostate and CG were observed between 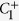 and 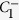, suggesting that the prostate dimensions were similar between PCa+ and Cl− subjects. For all subpopulations 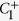, 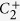, 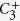, 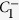, 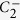, 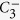, *C*^+^ and *C*^−^ the mean prostate and CG volumes after atlas construction appeared to be smaller compared to prior to atlas construction since the atlas tends to shrink the prostate when aligning multiple glands into a common frame of reference.

### Comparing prostate and central gland shape between PCa+ and PCa− subpopulations

**Figure 1:**
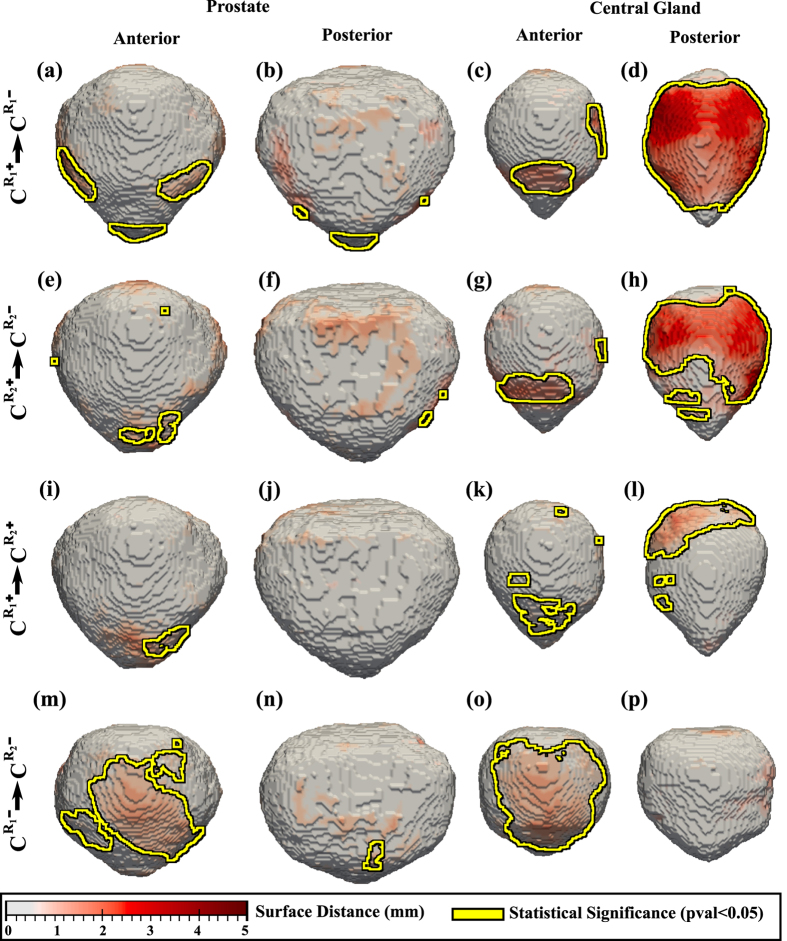
Shape differences between subpopulations illustrated as a distance map between the shape of the prostate (columns 1–2) and CG (columns 3-4). Yellow outlines show statistically significantly different regions when comparing (**a-d**) 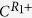 with 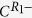 constructed using the delineations of rater 1; (**e–h**) or 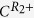 with 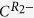 constructed using the delineations of rater 2, (**i–l**) 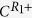 with 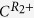, and (**m–p**) 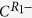 with 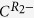.

**Figure 2:**
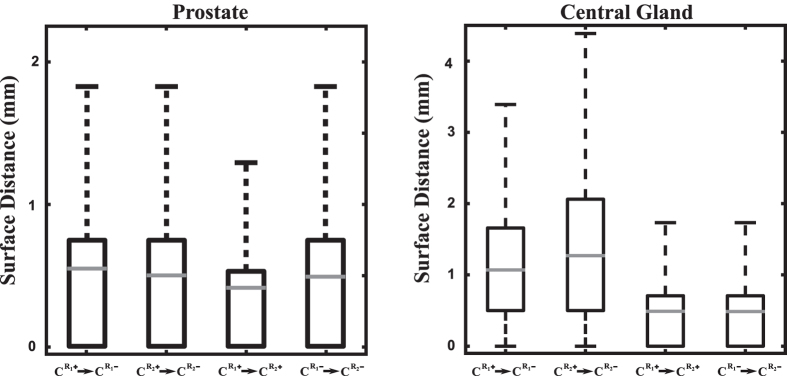
Surface distances between prostate (left) and CG (right) atlases when comparing 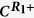 with 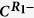, 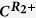 with 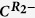, 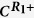 with 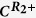, and 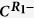 with 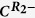.

**Figure 3:**
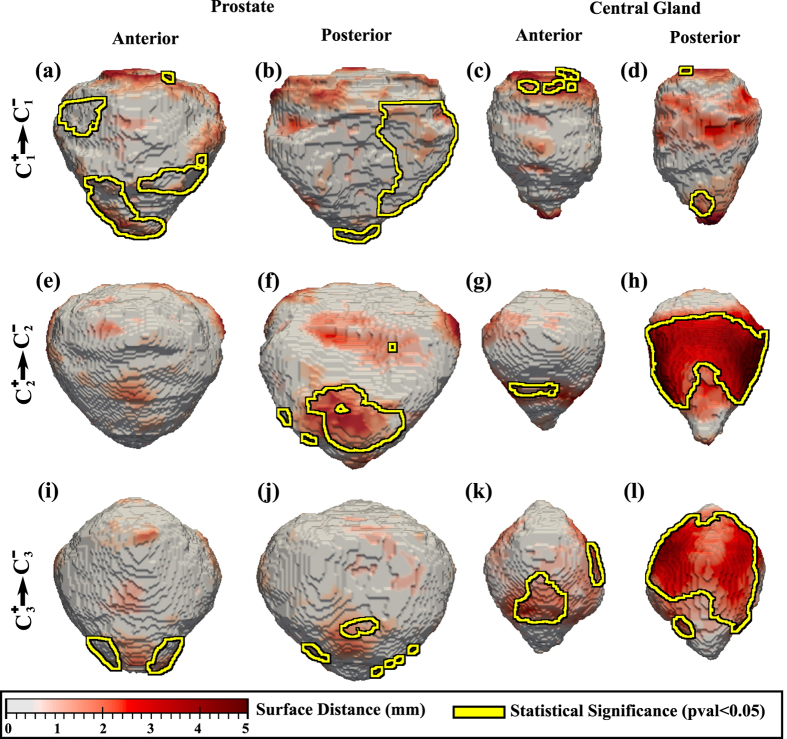
Shape differences between subpopulations illustrated as a distance map between the shape of the prostate (columns 1-2) and CG (columns 3-4). Yellow outlines show statistically significantly different regions when comparing (**a**–**d**) 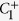 and 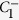, (**e**–**h**) 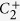 and 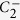, (**i**–**l**) 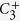 and 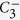.

Multiple comparisons were performed to assess the inter-rater variability (Fig. 1) and to characterize the differences in the prostate and CG shapes on account of the presence of cancer (Figs 1, 2 and 3).

The comparison of *C*^+^ and *C*^−^ revealed shape differences on the anterior side of the prostate (Fig. 1a,b,e,f). Statistically significant differences were also apparent within the CG on the posterior side (Fig. 1d,h), between *C*^+^ and *C*^−^ subpopulations, in some areas the differences being as large as 5 mm. These CG shape differences may be the outcome of the overall volume differences in CG (Table 2), possibly on account of the statistically significant increased CG volume in the Bx− subjects.

Across raters, similar trends are observed for the CG, when comparing *C*^+^ and *C*^−^ (Fig. 1c,d,g,h). To further assess the effect of the raters on the resulting atlases, we compared 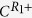 with 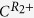 and 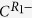 with 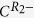 (Fig. 1i–p). Across PCa+ subjects, largest differences were observed on CG in the proximity of the base (Fig. 1l), while across PCa− subjects, differences were observed both on the prostate and CG on the anterior side (Fig. 1m,o). Although some of these differences were statistically significant, the magnitude of the difference observed when comparing 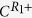 with 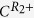 or 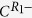 with 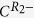 were considerably smaller (at most 2 mm), compared to the distances observed between 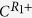 with 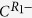 or 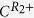 with 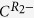, which were as high as 5 mm (Fig. 2).

The comparison of the 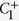 with 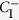 subpopulations revealed statistically significant differences in the shape of the prostate on both the anterior and posterior side, proximal to the apex (Fig. 3a,b). CG shape differences were also observed between 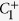 with 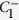 next to the inferior and superior side of the prostate (Fig. 3c,d). The PCa+ and PCa− comparison of the prostate and CG shapes within the *C*_2_ and *C*_3_ subpopulations appears to show a similar trend. Statistically significant differences in the shape of the prostate were also observable at the apex of the prostate (Fig. 3e,f,i,j), as also differences in the shape of the CG between the PCa+ and PCa− subjects, possibly on account of the enlargement of the CG in the Bx− subjects in 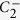 and 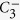.

**Figure 4:**
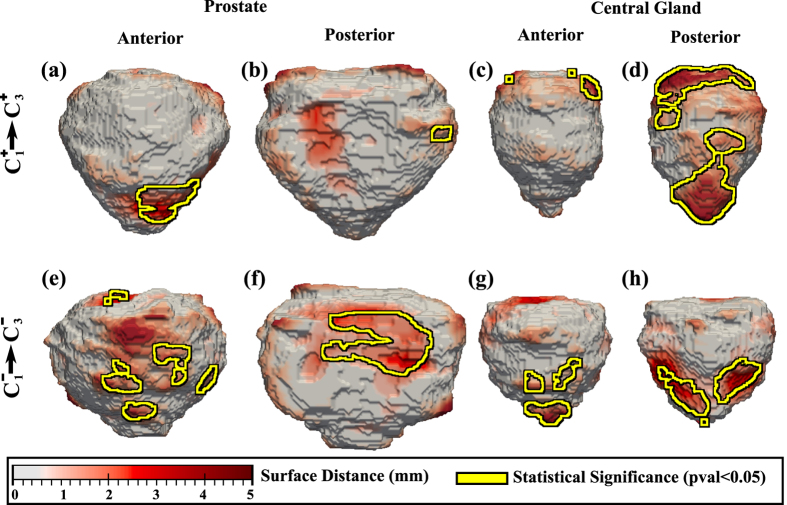
The distance maps show shape differences in prostate (**a**,**b**,**e**,**f**) and CG (**c**,**d**,**g**,**h**) when comparing (**a**–**d**) 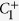 and 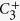 subpopulations, and (**e**–**h**) 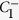 and 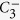 subpopulations (statistically significant differences outlined in yellow).

Finally, we also evaluated the variability in the shape of the prostate and CG when comparing 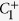 with 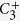 and 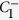 with 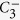, respectively. Only data from *C*_1_ and *C*_3_ was included in this comparison, as the MRI at these two institutions was acquired with a surface coil, as opposed to *C*_2_ for which an endorectal coil was used. The comparison of the 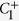 and 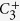 subpopulations showed localized statistically significant shape differences on the prostate next to the apex (Fig. 4a), and considerable differences on the posterior side of the CG (Fig. 4d). These differences appear to be related to the volume of CG which appears to be reduced in 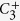. The comparison of the Cl− subpopulation in 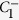 with the Bx− subjects in 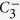 reveals shape differences in the prostate, localized in the mid-gland both on the posterior and anterior side (Fig. 4e,f). Moreover, CG shape differences are mostly localized in the inferior and superior extremity.

## Discussion

Despite the large variability in image acquisition parameters and protocols for the considered cohorts (Table 3), shape differences in the prostate were consistently observed between *C*^+^ and *C*^−^ subpopulations on the anterior side of the prostate. These differences do not appear to be on account of the inter-rater variability which was determined to be small. Shape differences between cancer positive (PCa+) and cancer negative (PCa−) men may be the result of cancer localized in the peripheral zone. Moreover, shape differences were observed on the posterior side of the CG (Fig. 3d,h). The inter-rater variability may indeed have affected the CG shape, but we believe that this contribution to shape differences between the population was a small part of the true anatomic differences manifest between PCa+ and Cl− subjects. The comparison of CG volumes (Table 2) shows statistically significant differences between Bx+ and Bx− subjects. Such differences in volume may induce changes in shape and might obfuscate shape changes induced by cancer presence. However, when comparing the 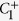 and 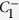 subpopulations, the Cl− subjects were found to have similar CG volumes compared to the Bx+ subjects, and had observable shape differences in the CG on the inferior side which cannot be attributed to the differences in CG volumes.

Our preliminary study appeared to suggest that for at least two different institutions, the Bx− patients had an increased prostate and CG volume when compared to the Bx+ subjects. Studies have suggested^18^ that the presence of benign prostatic hyperplesia in Bx− subjects could be a contributing factor for the increased prostate volume observed in the biopsy negative prostates. Moreover, large prostates naturally produce more PSA compared to smaller glands^19^, resulting in a considerable number of enlarged prostates in the Bx− subpopulation. To the best of our knowledge, the patients considered in the Bx− subpopulations were truly prostate cancer negative, as they underwent MRI-US fusion biopsy and had 12+ negative biopsy cores.

The CG shape difference between 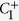 and 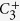 subpopulations were localized to the base of CG on the posterior side of the prostate. Although the localization of PCa was not accurately known for the Bx+ patients in none of the three institutions 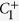, 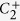 and 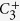, the observed variability in the shape of the CG could be attributed to the possible presence of prostate cancer in the peripheral zone, distending the border between the CG and the peripheral zone^4^. Moreover, our study revealed that the differences in the shape of the prostate and CG between the patients in 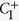 and 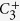 were different compared to the trends observed in comparing the *C*^+^ and *C*^−^ subpopulations, suggesting that the presence of prostate cancer appears to alter the normal shape anatomy of the prostate and the CG.

Our study did have its limitations. This was a preliminary study comprising a relatively small number of subjects. Also, the comparison of prostate shapes between subpopulations is a difficult task on account of various factors. For instance, the prostate shape varies naturally and changes with age as the volume of the prostate increases^19^. The atlas comparison framework was designed to correct the natural variability and allow the comparison of shapes. Moreover, segmenting the prostate and CG can be challenging, especially at the base and apex of the prostate^20^. In the current study, two expert raters segmented the prostate and the CG. Our study assessed inter-reader variability and its influence on the detected shape differences between PCa+ and Cl− subjects. An additional limitation was the lack of precise knowledge of spatial location of cancer presence within the prostate for the cases considered in this study. One of the aspects we wish to explore in future work is to carefully and precisely correlate the spatial location of the cancer with the site of observed shape differences.

The goal of this exploratory study was to characterize and evaluate the presence of shape differences in the prostate and central gland on T2w MRI between patients with and without prostate cancer. Statistically significant differences were observed in all three institutions from which cases were acquired for this study on the posterior side of the prostate between patients with and without prostate cancer. Similarly, differences were observed on the central gland at the boundary with the peripheral zone between patients who had negative and positive TRUS-MRI fusion prostate biopsies across two different institutions. These differences will need to be independently validated in future studies.

## Material and Methods

### Data

**Figure 5:**
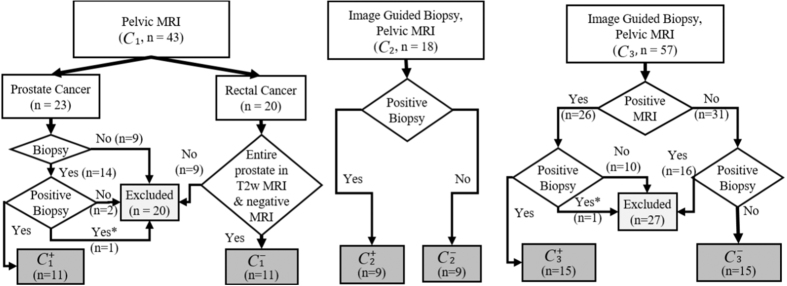
Inclusion criteria for the three institutions. *Indicates patients who were excluded in *C*_1_ and *C*_3_ to generate sized match populations.

Our study included 70 subjects (Fig. 5) with a pelvic MRI exam (Tables 1 and 3). The scans were acquired at three different institutions. Each institution employed a MRI scanner from a different vendor (Siemens, GE, Philips). However, the scans were done at the same magnetic field strengths (3 Tesla) using a Spin Echo T2w Sequence. The echo and repetition times varied across scanner platforms.

The cancer positive group, *C*^+^ included 35 Bx+ subjects: 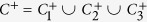. *C*^−^ included 11 prostates (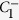) from subjects without clinical indication of PCa, who had a pelvic MRI acquired for rectal cancer diagnosis and a negative prostate MRI. There were an additional 24 Bx− in 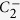 and 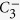: 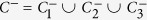. The same number of subjects were considered from each institution in *C*^+^ and *C*^−^. With the exception of the imaging studies acquired as part of the rectal diagnostic exam, each pelvic MRI was acquired either as part of an initial prostate pre-biopsy scan or as part of an active surveillance protocol for following low risk prostate cancer patients with imaging. The accurate location of the prostate cancer was not definitively known for the *C*^+^ studies, since radical prostatectomy specimens were not available for all subjects in this study.

Two expert raters annotated the boundary of the prostate and CG on T2w MRI using either MeVisLab^21^ or 3D Slicer^22^. Although the T2w intensity distrubutions within the prostate might vary depending on the choice of scanner platform and acquisition parameters, the shape of the prostate and CG as one might expect, is more resilient to acquisition variations compared to the original MRI signal intensity. Bias-field correction^23^ was applied to the endorectal MRI scans to reduce the artifacts manifesting as hyper-intense signal around the coil.

### Ethics Statement

Data analysis was waived review and consent by the IRB board, as this study included retrospective data that was deidentification. All experimental protocols were approved under the IRB protocol #02-13-42C with the University Hospitals of Cleveland Institutional Review Board, and all experiments were carried out in accordance with approved guidelines.

### Atlas Construction

The atlas construction framework was previously described by us in ref. 15. Below, we provide a brief summary of the approach. For details on the methodology and the validation of the atlas construction framework, we refer the interested reader to ref. 15.

For the atlas construction, each subject is defined in 3D by the axial T2w MRI exam, along with the outlines of the prostate and CG. The atlas construction for any subpopulation, involves spatially aligning the image scans for all the patients within that subpopulation. Once aligned, the scans from all subjects were used to create the statistical atlas which has three components: a T2w intensity atlas, a prostate shape atlas and a CG shape atlas. The core of the atlas construction approach involves identifying the optimal transformation of each subject MRI scan to align with the scans already embedded within the atlas. This process therefore forces all the patient scans within a subpopulation to be spatially aligned with respect to each other during the atlas construction. Specifically, the anatomically constrained registration scheme was employed for the atlas construction^15^ as it imposes constraints on the allowable deformation of the entire prostate and the CG, thereby preventing unrealistic deformations. The registration involves alignment of each subject to the atlas by optimizing a scoring function involving three terms: an intensity term which seeks to align the T2w intensity profiles, a prostate shape and a CG shape term which ensure the alignment of the prostate and CG shapes for each of the subjects with respect to the atlas. The prostate and CG shapes have the same weight in the scoring function. The construction framework results in shape atlases for both the entire prostate and CG which capture variations in shape and volume within the respective subpopulations.

The construction framework employs an iterative optimization approach to identify the transformation of each subject MRI. At each iteration, the complexity of the optimized transformation increases. At the end of each iteration, the atlas is updated by averaging the scans from all subjects previously aligned to the atlas. To obtain the statistical shape atlas of the prostate, the prostate boundary of each subject is represented as a signed distance function with respect to the prostate surface and averaged to create a statistical shape model of the prostate. Similarly, the CG shape atlas is constructed by averaging the signed distance representation of the CG model outlines for each subject. The updated atlas then employed as a baseline reference for registration for the subsequent iteration. The transformations applied to each subject’s MRI scan during atlas construction are described below.

- First, each subject MRI scan is translated to the common space. The delineated prostate and CG volumes are isotropically scaled to a common prostate volume within the atlas. The common prostate volume represents the median of the volumes of all the prostates within the subpopulation.
- Next, an affine transform is optimized to enable the identification of the translation, rotation and scaling operations needed to align the prostate and CG for each subject to the atlas. The atlas is updated with the transformed MRI from each subject.
- Finally, an elastic deformation is optimized to ensure the alignment of the prostate and CG of each subject relative to the prostate and CG of the atlas. The final atlas is updated by applying the optimal elastic alignment to each of the individual subject scans within the subpopulation.

Atlases were constructed for *C*^+^ and *C*^−^ as well as each of the subpopulations 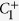, 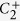, 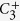, 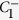, 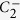 and 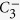. These atlases allowed for the study of shape differences.

### Atlas Statistical Comparison

**Figure 6:**
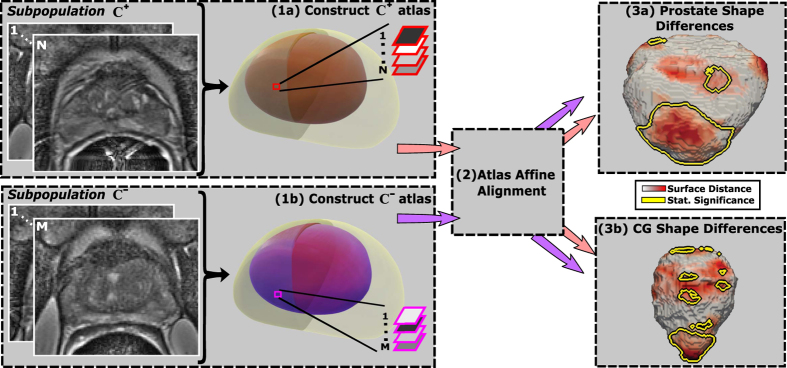
Framework for atlas comparison for two subpopulations, *C*^+^ and *C*^−^; (1) Statistical shape atlases are constructed for (**a**) *C*^+^ and (**b**) *C*^−^ for the entire prostate (grey, purple) and CG (red, purple); (2) The *C*^+^ and *C*^−^ atlases are aligned to a common space; (3) per-voxel statistical comparisons are performed to assess shape differences on the prostate boundary and CG.

The *C*^+^ and *C*^−^ atlases were quantitatively compared to characterize shape differences in the prostate boundary and CG (Fig. 6). The *C*^+^ atlas is constructed independently of the *C*^−^ atlas which may cause a possible translational and rotational shift of the *C*^+^ and *C*^−^ atlases relative to each other, and hence we first correct these linear differences. Module 2 focuses on identifying the optimal affine transformation to align *C*^+^ and *C*^−^ into the same common space (Fig. 6). Finally, statistical comparisons using the non-parametric Wilcoxon tests^24^ were applied for each voxel within *C*^+^ and *C*^−^ to identify shape differences on the prostate boundary and the CG (Modules 3a,b). The anatomic shape, e.g CG, is represented as a distance map, where each voxel in the volume reflects its corresponding distance to the surface. As opposed to the binary representation in which each voxel within CG is 1, and 0 outside of CG, the distance map provides a continuous quantitative representation of CG which facilitates statistical comparisons. Since neighboring voxels may be correlated, we assessed the statistical significance of shape differences between *C*^+^ and *C*^−^ after correcting for multiple comparisons using the Bonferonni method^25^. Hence, a voxel was considered as belonging to a region exhibiting statistically significant differences between shapes, if the p-value estimated by the Wilcoxon test was less than 0.05, the p-value being divided by the number of voxels associated with both the prostate and CG boundaries of the atlas being evaluated.

Here, we included annotations of the CG and prostate from two expert readers and constructed various atlases to evaluate inter-rater variability and its influence on the shape differences observed between PCa+ and PCa− prostates. Specifically, we constructed the *C*^+^ and *C*^−^ atlases employing the delineations from each rater. 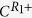 and 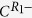 denote the atlases constructed for the PCa+ and, respectively, PCa− subjects using the delineations of the prostate and CG obtained from rater 1. Similarly, the delineations of rater 2 were utilized to construct 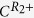 and 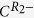. When not explicitly referencing the raters, we used *C*^+^ and *C*^−^ in place of 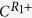 and 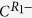. Similarly, the subpopulations atlases, 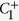, 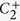, 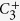, 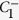, 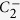 and 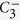, were constructed using the delineations of rater 1.

Our evaluation assessed the differences in the shape of the prostate and CG that are the results of the inter-rater variability across subpopulations when stratified by disease type. We aimed at comparing the extent and the location of shape differences as a result of inter-rater variability (assessed by comparing 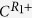 with 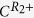, and 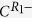 with 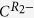) with the shape differences observed between PCa+ and PCa− subjects (resulting from comparing 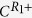 with 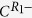, and 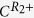 with 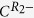). Our hypothesis is that inter-rater variability is substantially smaller compared to the true anatomic differences manifest between PCa+ and Cl− subjects.

### Evaluation

The anatomic shape of the prostate and CG in a given subpopulation can be affected by 1) the characteristics of patient population included in this study, and 2) the extent and volume of disease present. During atlas construction, their shape is affected by the ability of the atlas construction framework to correct for shape variations within the subpopulation. To identify the shape alterations induced by the presence of disease, one first needs to control for the natural variations intrinsic to each subpopulation.

To assess the ability of the atlas to correct for the natural variability of the prostate anatomy within each subpopulation, we evaluated the volume of the entire prostate and the CG before and after atlas construction. Specifically, we attempted to evaluate whether the variation in volumes was also correlated with shape variability of the prostate and CG within each subpopulation. The atlas construction framework is expected to reduce the standard deviation of these volumes, since the MRI scans and linked prostate and CG volumes of all subjects are aligned to each other. Thus, a smaller standard deviation should be observed after atlas construction. Moreover, we used the Wilcoxon test to identify whether there were statistically significant differences between the prostate and CG volumes when comparing PCa+ and PCa− subpopulations. Statistically significant differences may suggest that volume differences still exist, and the resulting shape differences may not necessarily be attributed to cancer presence since the prostate and CG volume could also affect their corresponding shape and appearance.

## Additional Information

**How to cite this article**: Rusu, M. *et al*. Computational imaging reveals shape differences between normal and malignant prostates on MRI. *Sci. Rep.* **7**, 41261; doi: 10.1038/srep41261 (2017).

**Publisher's note:** Springer Nature remains neutral with regard to jurisdictional claims in published maps and institutional affiliations.

## Figures and Tables

**Table 1:** Prostate and CG volumes evaluated across the different cohorts, *C* ^+^, *C* ^−^, 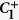 , 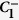 , 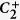 , 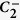 , 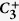 , 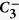 , and across the two raters.

Rater	Volume (ml)¶	Total			*C*_1_			*C*_2_			*C*_3_		
		*C*^+^	*C*^−^		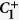	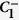		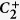	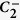		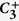	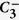	
Rater 1	Prostate	42.1 ± 18.5	60.6 ± 32.0	**0**.**01**	40.1 ± 21.0	32.6 ± 14.6	0.44	41.1 ± 13.4	79.5 ± 32.9	**<0**.**01**	44.2 ± 20.2	69.8 ± 27.9	**0**.**01**
Rater 2	Prostate	41.4 ± 18.3	60.0 ± 33.4	**0**.**01**	39.2 ± 20.6	29.9 ± 12.1	0.3	40.9 ± 13.6	81.5 ± 35.3	**<0**.**01**	43.3 ± 20.0	69.2 ± 28.1	**<0**.**01**
Rater 1	CG	17.8 ± 13.2	34.4 ± 24.6	**<0**.**01**	20.2 ± 17.0	16.6 ± 9.5	0.95	15.9 ± 6.5	48.0 ± 29.2	**<0**.**01**	17.1 ± 12.4	39.2 ± 22.9	**<0**.**01**
Rater 2	CG	18.0 ± 13.2	34.7 ± 26.7	**<0**.**01**	20.0 ± 17.1	15.2 ± 9.8	0.70	16.0 ± 6.5	50.7 ± 33.9	**<0**.**01**	17.8 ± 13.7	39.3 ± 22.9	**<0**.**01**

Bold font indicates significant p values < 0.01.

^¶^Mean ± standard deviation.

**Table 2:** Prostate and CG mean volumes after atlas construction.

Cohort	Region	Volume¶	Cohort	Region	Volume¶	Pval
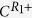	Prostate	34.8 ± 6.0	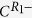	Prostate	36.5 ± 5.6	0.49
	CG	13.4 ± 2.1		CG	18.5 ± 3.5	**<0**.**01**
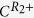	Prostate	35.2 ± 6.2	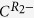	Prostate	36.9 ± 5.8	0.52
	CG	13.9 ± 2.2		CG	18.5 ± 3.7	**<0**.**01**
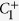	Prostate	32.2 ± 4.5	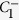	Prostate	31.9 ± 6.8	0.70
	CG	13.9 ± 3.0		CG	14.6 ± 3.7	0.51
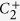	Prostate	36.2 ± 9.2	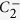	Prostate	39.7 ± 3.9	0.67
	CG	13.7 ± 1.9		CG	22.6 ± 3.5	**<0**.**01**
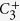	Prostate	39.7 ± 3.5	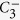	Prostate	40.4 ± 3.4	0.95
	CG	13.9 ± 2.8		CG	21.1 ± 4.5	**<0**.**01**

Bold font indicates significant p values < 0.01.

^¶^Mean ± Standard deviation, ml.

**Table 3:** Patient characteristics.

Variable	Total			*C*_1_			*C*_2_			*C*_3_		
	*C*^+^	*C*^−^	Pval	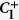	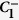	Pval	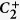	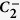	Pval	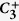	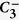	Pval
	n = 35 (50.0%)	n = 35 (50.0%)		n = 11 (15.7%)	n = 11 (15.7%)		n = 9 (12.8%)	n = 9 (12.8%)		n = 15 (21.4%)	n = 15 (21.4%)	
Coil Type				Surface	Surface		Endorectal	Endorectal		Surface	Surface	
Pixel§	192–1024	240–1024		192–320	240–320		512	512		1024	1024	
Resolution†	0.21–0.97	0.21–0.9		0.37–0.97	0.50–0.90		0.35	0.35		0.21	0.21	
Sl. Spacing‡	1.5–3.5	3.0–3.4		1.5–3.0	3.0–4.0		3.0–3.5	3.0–3.5		3.0–3.4	3.0–3.4	
Age, years¶	63.8 ± 8.6	61.0 ± 10.6	0.17	62.5 ± 7.8	62.6 ± 17.3	0.79	60.6 ± 10.5	62.0 ± 6.8	0.96	66.7 ± 7.33	59.3 ± 5.0	**<0**.**01**
PSA, ng/ml¶	7.6 ± 5.1	5.8 ± 2.1	**<0**.**01**	8.6 ± 5.75	NA		7.85 ± 3.5	6.35 ± 2.6	0.65	6.8 ± 5.5	5.4 ± 2.1	1.00
Stage, range	T1c–T3	NA		T1c–T2a	NA		T1c–T2c	NA		T1–T3	T1–T2	
Gleason*	(5, 26, 2, 2)	NA		(2, 8, 1, 0)	NA		(3, 5, 1, 0)	NA		(0, 13, 0, 2)	NA	

Bold font indicates significant p values < 0.01.

NA, non-available.

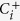
, cancer positive subjects, cohort i = 1..3.

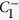
, subjects without clinical indication of prostate cancer and with a negative MRI, cohort 1.

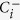
, biopsy negative subjects, cohort i = 2..3.

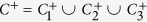
.

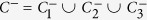
.

^§^In plane pixel count, range.

^†^In plane resolution, (mm).

^‡^Slice Spacing, range (mm).

^¶^Mean ± standard deviation.

^*^Gleason Grade Counts (3 + 3, 3 + 4/4 + 3, 4 + 4, 4 + 5).
